# Validation of inertial measurement units with optical tracking system in patients operated with Total hip arthroplasty

**DOI:** 10.1186/s12891-019-2416-4

**Published:** 2019-02-06

**Authors:** Roland Zügner, Roy Tranberg, John Timperley, Diana Hodgins, Maziar Mohaddes, Johan Kärrholm

**Affiliations:** 1 Department of Orthopedics, Institute of Clinical Sciences, Sahlgrenska Academy,University of Gothenburg, Sahlgrenska University, 413 45 Göteborg, SE Sweden; 2 Exeter Hip Unit, Princess Elizabeth Orthopaedic Centre, Royal Devon & Exeter NHS Foundation Trust, Exeter, EX2 5DW UK; 3 European Technology for Business Ltd, London, UK; 4 Lundberg Laboratory for Orthopaedic Research, Sahlgrenska University Hospital, Gröna stråket 12, SE-41345 Göteborg, Sweden

**Keywords:** Inertial measurement units, IMU’s, Optical tracking system, Total hip arthroplasty, Gait, Gait analysis

## Abstract

**Background:**

Patient reported outcome measurement (PROMs) will not capture in detail the functional joint motion before and after total hip arthroplasty (THA). Therefore, methods more specifically aimed to analyse joint movements may be of interest. An analysis method that addresses these issues should be readily accessible and easy to use especially if applied to large groups of patients, who you want to study both before and after a surgical intervention such as THA. Our aim was to evaluate the accuracy of inertial measurement units (IMU) by comparison with an optical tracking system (OTS) to record pelvic tilt, hip and knee flexion in patients who had undergone THA.

**Methods:**

49 subjects, 25 males 24 females, mean age of 73 years (range 51–80) with THA participated. All patients were measured with a portable IMU system, with sensors attached lateral to the pelvis, the thigh and the lower leg. For validation, a 12-camera motion capture system was used to determine the positions of 15 skin markers (Oqus 4, Qualisys AB, Sweden). Comparison of sagittal pelvic rotations, and hip and knee flexion-extension motions measured with the two systems was performed. The mean values of the IMU’s on the left and right sides were compared with OTS data.

**Results:**

The comparison between the two gait analysis methods showed no significant difference for mean pelvic tilt range (4.9–5.4 degrees) or mean knee flexion range (54.4–55.1 degrees) on either side (*p* > 0.7). The IMU system did however record slightly less hip flexion on both sides (36.7–37.7 degrees for the OTS compared to 34.0–34.4 degrees for the IMU, *p* < 0.001).

**Conclusions:**

We found that inertial measurement units can produce valid kinematic data of pelvis- and knee flexion-extension range. Slightly less hip flexion was however recorded with the inertial measurement units which may be due to the difference in the modelling of the pelvis, soft tissue artefacts, and malalignment between the two methods or misplacement of the inertial measurement units.

**Trial registration:**

The study has ethical approval from the ethical committee “Regionala etikprövningsnämnden i Göteborg” (Dnr: 611–15, 2015-08-27) and all study participants have submitted written approval for participation in the study.

## Background

Osteoarthritis (OA) is a chronic joint disease and the World Health Organization report that 10% of all men and 18% of all women aged over 60 years have a symptomatic osteoarthritis and 80% of those with osteoarthritis have affected joint movements [1]. Total hip arthroplasty (THA) is a common treatment for patients diagnosed with hip osteoarthritis when non-surgical treatment has failed. In Sweden, approximately 17,000 THA are performed every year and the majority of these are due to primary osteoarthritis. According to the Swedish Hip Arthroplasty Register (SHAR) most of the patients (89%) report that they are satisfied with the result one year after their hip operation. However, the remaining 11% report that they are unsatisfied or less satisfied with the operation. They report problems with pain, anxiety and depression, and movement. Recordings of joint movements before and after THA could be of value to estimate the potential efficacy of the procedure and to document any remaining restrictions of motions with potential impact on the clinical outcome in a large group of patient with osteoarthritis. Recordings of remaining gait restrictions may be used as quantitative results of the surgical intervention and may also be of value in the planning of any further treatment or rehabilitation in those patients who still experience motion restrictions [1–16]. Furthermore, hip range defines the stride length of the patient, which is a parameter used when assessing mobility.

Ewen et al. reviewed seven studies of gait analysis performed after insertion of a THA. Three of these studies found a significantly decreased walking speed in the studied group compared to controls, a reduced stride length and a reduced range of hip extension-flexion. The authors concluded that speed, stride length, range of hip extension-flexion seemed to be key variables that differentiate a population of THA from controls [17] .

The ability to transfer images into computers using non-invasive optical tracking systems (OTS) normally used in modern gait analysis has enabled them to be used for evaluation studies after total replacement of the hip joint. The OTS method uses retro-reflective markers attached to the skin, tracked with high speed video cameras together with a system for data analysis and calculations of joint motions. The retro-reflective markers used may be individually attached to the skin to facilitate data capture of the recordings; the technique is frequently used in clinical practice with different marker protocols [18–32]. A second way to measure joint motions after different interventions, especially during gait, is to use inertial measurement units (IMUs). These devices incorporate three orthogonal accelerometers and gyroscopes and may also include magnetometers. The major advantage of using IMUs is that it is possible to use these devices in different environments and less time is required to perform the examination. Furthermore, no cameras and force plates need to be used which makes the system more flexible and suitable for monitoring joint movement. So far most studies of joint kinematics have been based on recordings with use of OTS. Validation of the IMU system with use of OTS as a standard reference is therefore relevant, as it will open up opportunities for all patients to be monitored in the clinic.

Our hypothesis is that the IMU system and OTS will record comparable values for the range of sagittal plane kinematics in the chosen joints. Our aim is to compare an IMU system with an OTS as reference, during walking, obtaining simultaneous recordings of pelvic, hip- and knee joint motions in patients who have received a total hip replacement.

## Methods

### Subjects

To obtain a representative material we wanted to include both patients who were satisfied, less satisfied or dissatisfied. During the period 2011–2013, 54 patients operated with a THA in the University Hospital NN had reported mobility problems 1 year postoperatively, 25 of whom accepted to participate in the study. A cohort of 25 patients with no reported mobility problems in the EQ. 5D form was also identified and included. The identification of the two cohorts was based on the results from the postoperative EQ. 5D questionnaires. One patient was excluded due to technical problems, which resulted in 25 males and 24 females analysed. Nineteen had been operated on the left and 30 on the right side and sixteen of the patients had also been operated on the contralateral side earlier.

At the latest operation the patients had a mean age of 71 years (51–80) and a body mass index (BMI) of 28.7 (20–44). The median time between the total hip arthroplasty and the gait investigation was 36 (22–56) months.

The study has ethical approval from the ethical committee “Regionala etikprövningsnämnden i Göteborg” (Dnr: 611–15, 2015-08-27) and all study participants have submitted written approval for participation in the study.

#### Experimental setup

**Fig. 1:**
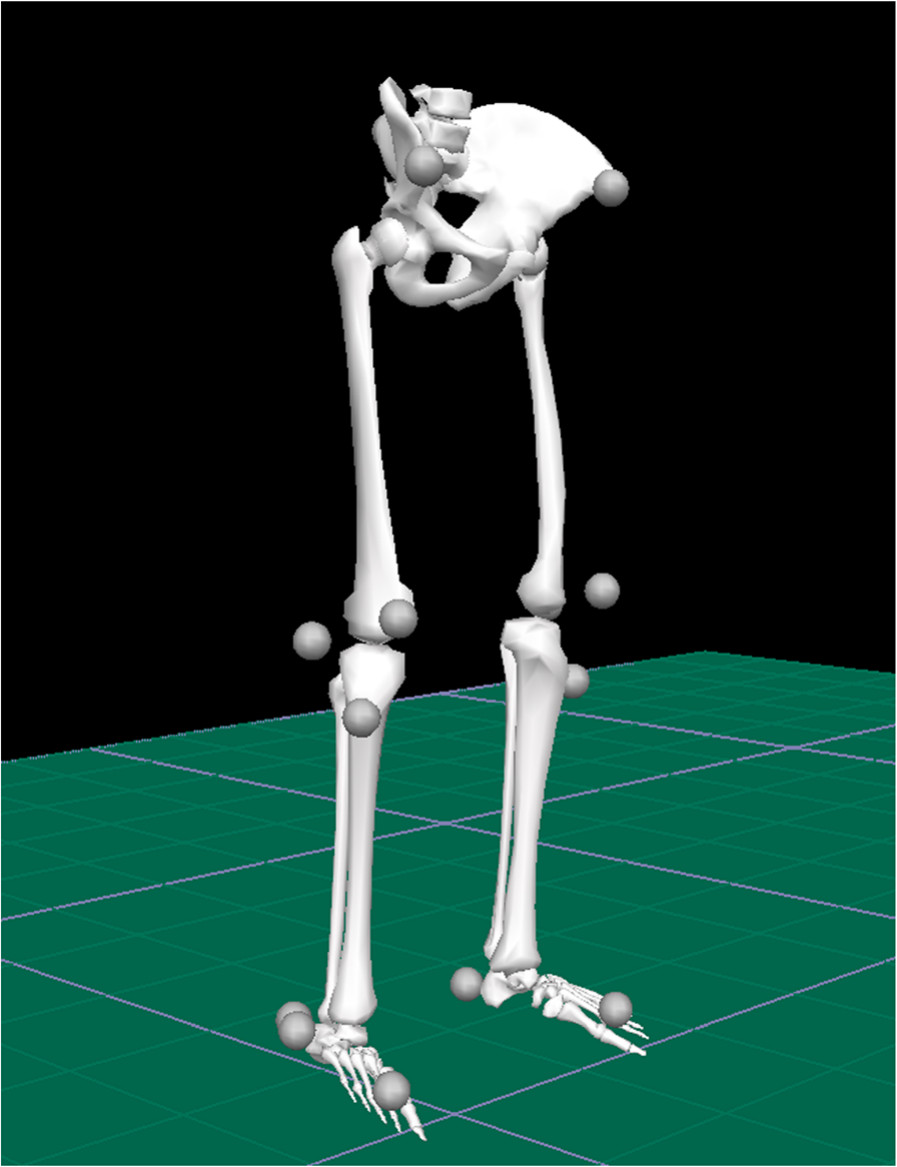
Stick figure

For data acquisition with the OTS system, a 12-camera motion capture system (Oqus 4, Qualisys AB, Göteborg, Sweden) was used to determine the positions of 15 skin markers (Fig. 1) which were attached to the proximal border of sacrum, anterior/superior of iliac spine, lateral knee joint line, proximal boarder of patella, tibial tubercle, tuber calcanei at the heel, lateral malleolus and finally between the second and third metatarsals which formed the marker model [33]. In the OTS the proximal segment was fixed and the distal segment the moving segment and the OTS calculations were based on Euler angles. The exposure rate of the OTS was 240 fps and the recorded marker data of the marker model were filtered using a Butterworth 4th order filter with a cut of frequency of 6 Hz.

For calculations of kinematic peak variables together with spatiotemporal gait parameters, the Visual 3D™ software (C-Motion, Inc., Germatown, USA) was used. A modified Coda pelvis based on the bilateral markers on anterior superior iliac spine together with one marker on the mid-point on the proximal border of sacrum was used to define the pelvis segment.

For the IMU system (GaitSmart™, Hertfordshire, United Kingdom) the joint angle was calculated by computation of the angle required to rotate one sensor on to the second sensor using an axis of rotation that is not constrained to a specific plane. The device measures sagittal plane and frontal plane motions, which will match those of the subject provided that it is correctly positioned in relation to anatomical axes. After calibration the IMU system measures angles in relation to an axis perpendicular to the floor (Global system). Proprietary software (Poseidon Version 9.1.4) transformed the raw data from the gyroscopes and accelerometers into angular position along the sensor axes, which align with the anatomical axes when correctly mounted.

The sampling rate was 102.4 Hz. Rotation in the transverse plane is not measured by the IMU and was thus excluded from our evaluation. The hip joint angle was determined by the pelvis and thigh sensors and for the knee joint the angle subtended by thigh and calf sensors.

The IMU model for the pelvis uses two sensors which will detect any measurable movements at the sacroiliac joints. The joint angle is the angle required to rotate the lower limb into alignment with the upper about the hinge axis in a right-handed rule. For timing of the two systems the initial contact on the first force-plate of the right foot was used. Recordings were performed simultaneously for the systems following two consecutive steps in the order of right and left. In the IMU system the angle measured corresponds to the combined angle in the sagittal and frontal planes, whereas the OTS is based on calculation of Eulerian angles and therefore more strictly measures flexion-extension as sagittal plane motions [34].

#### Subject’s preparation

**Fig. 2:**
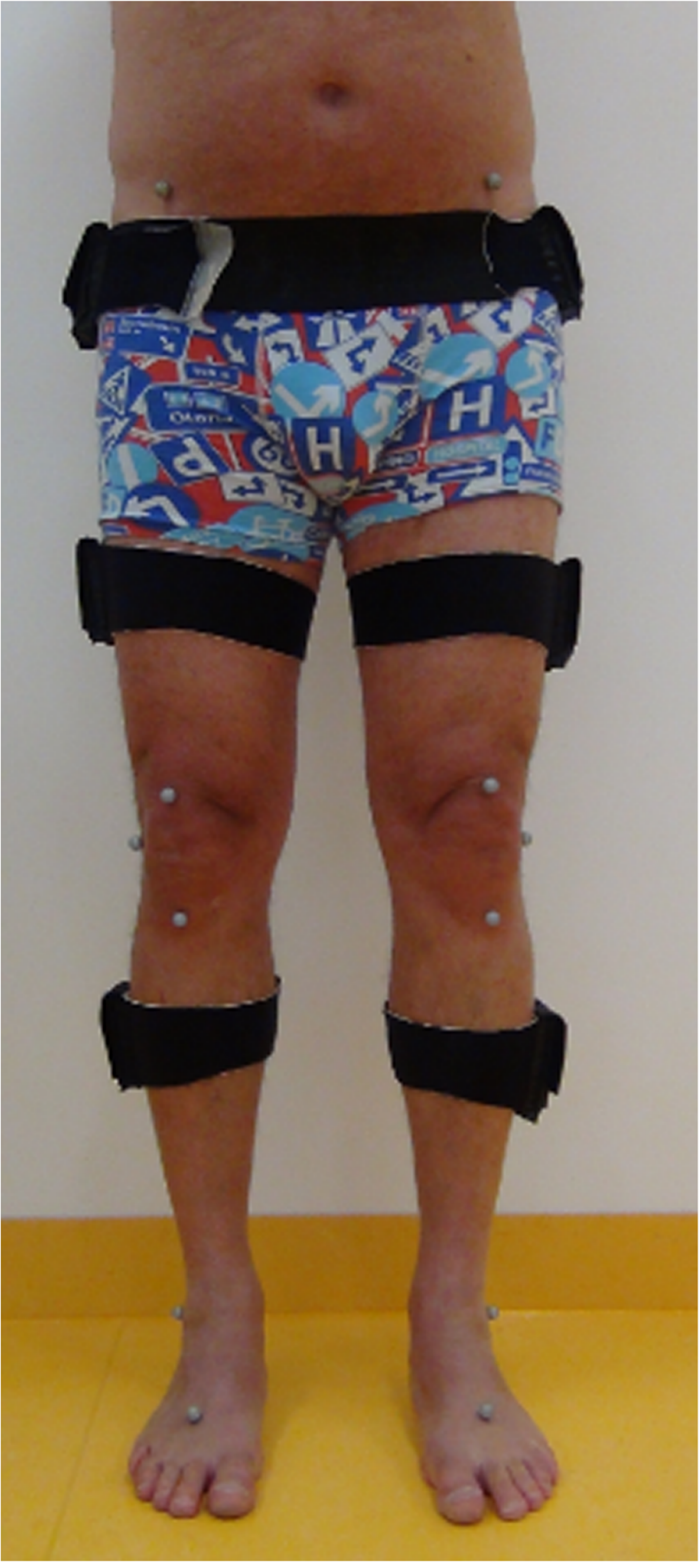
Front view IMU-sensors and OTS-markers

**Fig. 3:**
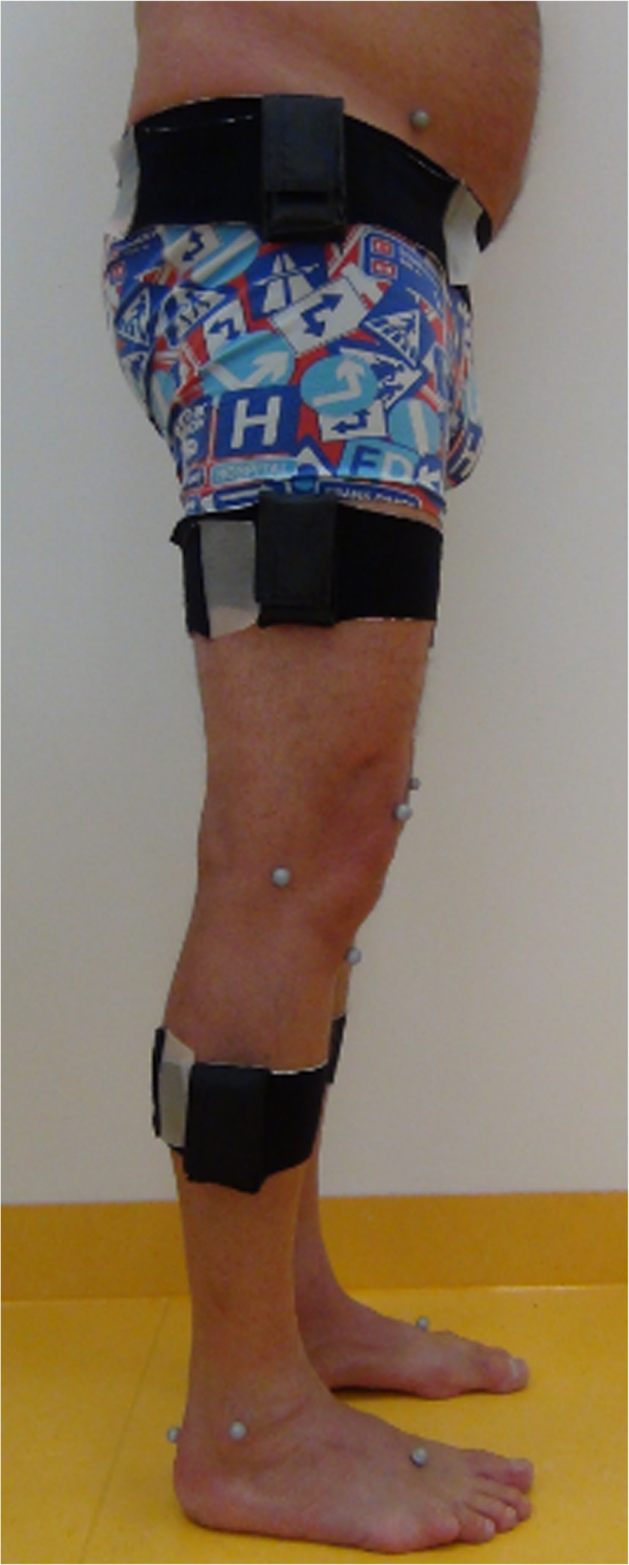
Side view IMU-sensors and OTS-markers

A physiotherapist with more than 15 years’ experience of OTS and marker placement positioned all markers and the 6 IMU sensors on all subjects. During the recording of data, the subjects were walking barefoot, wearing only underwear. Fifteen skin-markers (∅ 12 mm) were attached with double-sided adhesive tape [33]. At the same time IMU’s (GaitSmart™, Hertfordshire, United Kingdom) consisting of six IMU units were placed bilaterally on the lateral aspect of pelvis, thighs and shanks (Figs. 2 and 3). For the pelvis, the IMU sensors were placed under iliac crest following the alignment of the pelvis, for the thighs and the shanks the IMU sensors were placed on the widest lateral aspect of each segment using elastic straps and aligned into a straight vertical line in order to facilitate segment movements in sagittal plane for both systems [28–32]. There are small movements in the coronal and transverse planes at the hip joint during walking, but in this evaluation only sagittal plane movement, which is the most clinically relevant for hip replacement patients, was compared. The entire range of motion for pelvic, hip and knee joint in the sagittal plane, during the same complete gait cycle, was extracted from both gait analysis systems for calculations. Prior to recordings, subjects were first asked to walk 5–10 times, at their self-selected speed, through the measurement area consisting of 6 m × 2 m, to familiarize themselves with the situation. Thereafter the recording was taken.

#### Statistical analysis

An exploration of the data set with the Shapiro-Wilks test revealed that the variables of the kinematic hip and knee extension-flexion range were normally distributed whereas the recorded range of pelvic motions were not. The concurrent validity of the IMU system with use of the OTS as a reference standard was evaluated with computation of the intraclass correlation coefficient (ICC) and Bland-Altman plots. Wilcoxon rank test was used to compare the calculated median values with use of the two methods.

## Results

The median pelvic tilt (OTS recordings: 4.5°, IMU recordings: 4.6°) and the range of knee flexion-extension (OTS right: 55.1°, left: 54.4°, IMU right: 54.9°, left: 54.4°) did not differ between the two methods on either side (*P* ≥ 0.75). Comparison of range of hip flexion-extension revealed that the IMU system recorded about 3° smaller values on both sides (2.8° on the right and 3.3° on the left side) than the OTS did (*P* < 0.001, Table 1).

**Table 1:** Gait parameters for optical tracking system (OTS) and inertial measurement units (IMU)

	OTS			IMU			Wilcoxon	Intraclass correlation
	Mean	95% C.I.	Median	Mean	95% C.I.	Median	*P*-value^#^	ICC (CI)^¤^
Pelvic range degree	5.4	4.5–6.3	4.5	4.9*	4.4-5.3	4.6	0.95	0.08 (−0.20–0.35)
Hip ext./flex range right degree	36.8	35.2–38.5	36.2	34.0	32.2–35.9	33.4	< **0.001**	0.75 (0.34–0.89)
Hip ext./flex range left degree	37.7	36.0–39.4	38.3	34.4	32.7–36.2	34.4	< **0.001**	0.73 (0.22–0.89)
Knee ext./flex range right degree	55.1	53.5–56.7	55.0	54.9	53.1–56.6	53.9	0.75	0,83 (0,72-0,90)
Knee ext./flex range left degree	54.4	52.8–55.9	54.2	54.4	52.8–56.0	54.9	0.69	0.86 (0.77–0.92)

*Mean of right and left side

^#^*P*-values refer to Wilcoxon sign ranks test between OTS and IMU

^¤^Intraclass correlation coefficient (ICC) and 95% confidence interval (CI)

**Fig. 4:**
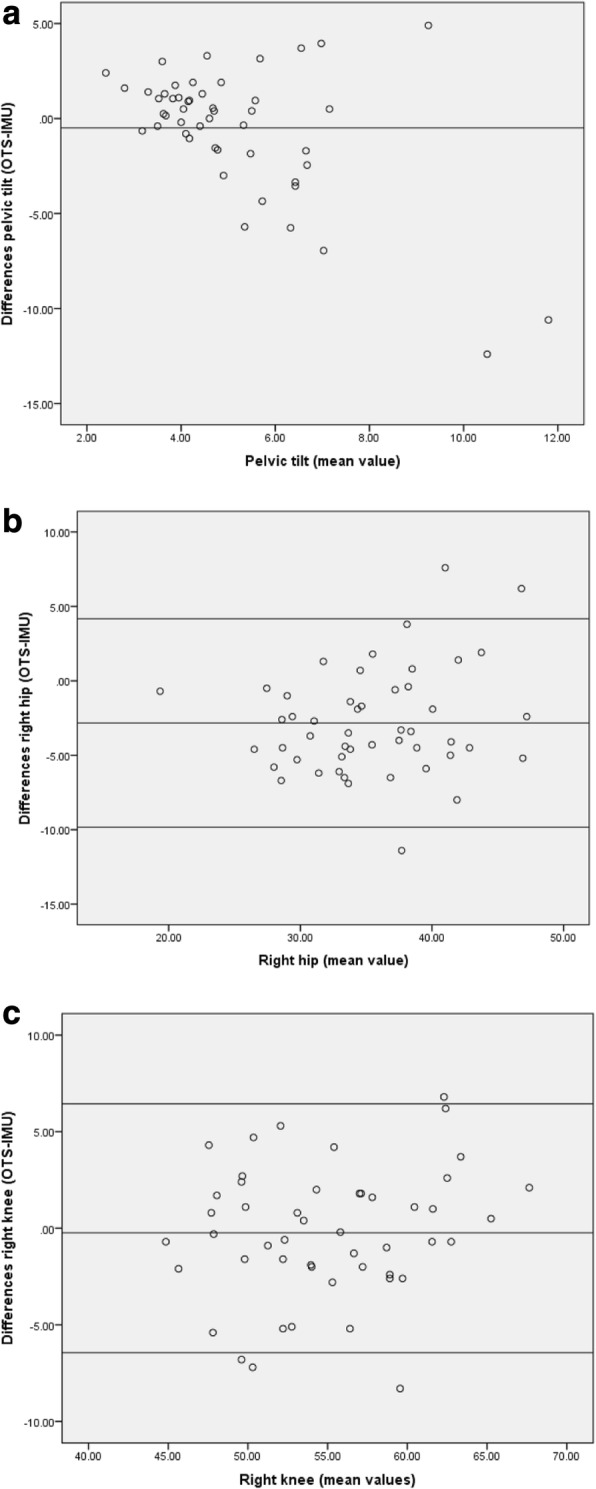
Bland-Altman plots showing to differences (y-axis) and mean values (x-axis), in pelvic tilt, right hip and right knee. **a**: The mean difference of pelvic tilt and 95% confidence interval of the difference was − 0.5 (− 1.5 to 0.5) **b**: The mean difference and 95% confidence interval of the difference of right hip flexion/extension range was − 2.8 (− 3.9 to − 1.8). The horizontal line above and below the mean are 1 standard deviation. **c**: The mean and 95% confidence interval of the difference of right flexion/extension range was − 0.24 (− 1.2 to 0.7). The mean difference and 95% confidence interval of the difference of right hip flexion/extension range was − 2.8 (− 3.9 to − 1.8). The horizontal line above and below the mean are 1 standard deviation

The intraclass correlation coefficient (ICC) and 95% confidence interval (CI) for the recordings of pelvic tilt were 0.08 and − 0.20-0.35. The range of pelvic tilt was wider according to the OTS (1.2–17.1° compared to 2.5–11.7° for the IMU). The mean difference of pelvic tilt was − 0.5 (95% confidence interval of the difference: − 1.5 to 0.5). The ICC values for the right and left hip flexion/extension range were 0.75 and 0.73 (95% CI: 0.34–0.89 and 0.22–0.89), respectively. The mean difference and 95% confidence interval of the difference of right and left hip flexion/extension range were − 2.8 (− 3.9 to − 1.8) and − 3.2 (− 4.3 to − 2.2) respectively. The ICC’s for right and left knee flexion/extension range were higher 0.83 and 0.86 (95% CI: 0.72–0.90 and 0.77–0.92, Table 1, Fig. 4). The mean differences and 95% confidence interval of the difference of right and left knee flexion/extension range were − 0.24 (− 1.2 to 0.7) and 0.01 (− 0.8 to 0.8) respectively.

### Discussion

We compared a gait analysis system based on 6 IMU sensors aligned to the lateral side of the pelvis and the lower leg with an OTS to evaluate the concordance between these methods when measuring pelvic, hip- and knee joint motions in the sagittal plane. We hypothesised that the IMU and OTS systems will record comparable values for the range of sagittal plane kinematics in the chosen joints. The pelvic segment was also monitored with each system, but with the OTS the pelvis was assumed to be a single fixed bone, whilst independent movement of the left and right pelvis IMUs were measured [35]. The reason for this is that in the IMU system left- and right-sided motions are calculated separately as an inherent property of this method. Comparison between the right and left sensors showed only a minor mean difference between the two sides.

In the comparison calculations of pelvic motions, the sagittal movement data from the IMU system was therefore averaged. However, for the hip calculation the left and right pelvis movement in the sagittal and coronal planes were used to calculate the left and right hip angle.

The comparison showed a small but statistically significant difference in the hip joint movement but not in pelvic and knee joint movements. However, it also showed high correlation for the hip and knee joints but not the pelvis. As the thigh sensors were common to the knee and hip angles, the pelvis is the likely cause for the small discrepancy between the two systems for the hip angle. This is further supported by the large spread in values for the pelvis for both systems, and the poor correlation between the OTS and IMU data for the pelvis. It is also recognised that the two systems use different method for calculating the hip angle and this may also add to the slight difference between the two systems (Table 1). In the IMU system the angle measured corresponds to the combined angle in the sagittal and frontal planes, whereas the OTS is based on calculation of Eulerian angles and therefore more strictly measures flexion-extension as sagittal plane motions.

The reliability of OTS has been performed previously [20–22, 25, 26, 36–38]. A systematic review of 15 papers provided an overview of the possible errors from an OTS in 2009 [22]. There findings showed that the mean precision error between and within assessors at measurements of hip- and knee joint flexion angles was less than 6°, in 12 studies*.* Based on these values the difference between OTS and IMU observed by us can be considered to be within the accuracy of OTS.

In 2010 Peters et al. performed a systematic review of 20 studies analysing the effect of soft tissue artefacts (STA) in the lower limb. In 13 studies invasive methods were used and it was reported that several factors could influence the results such as: the location of markers, the activity performed, the segment used and other characteristics of the individual. Up to 40 mm STA have been reported at the thigh and there is need for improved methods to increase the resolution [23]. The IMU system can be expected to be subjected to mounting errors, but according to our knowledge any comparison with true skeletal motions have so far not been made using this device.

In a previous study we compared OTS measurement of hip flexion with dynamic radiostereometric analysis (RSA) [38]. In that study the OTS system recorded lower flexion than RSA during the early phase of flexion. This observation suggests that IMU records still lower values than OTS compared to true skeletal movements. Further studies of combined RSA and IMU measurements are however necessary to further elucidate this question.

It must be noted that unlike the OTS system, IMU based systems all use their own algorithms. Thus, IMU and OTS comparisons studies refer to specific systems. McCarthy et al. used the same system as in present study to compare OTS with the IMU system to measure knee flexion range. The conclusion was that there was no statistical difference between the two systems, which supports the findings of this study [39].

In 2014 Leardini et al. performed a reliability and validity study of an IMU system (Riablo™; Trento, Italy) using OTS [30]. The accuracy was tested in 17 healthy subjects with 5 different rehabilitations exercises which were repeated twice including re-mounting the IMU’s. OTS was used simultaneously to record thorax- and knee flexion angels in sagittal plane with attached reflective markers. Thoracic motions were measured in relation to the laboratory coordinate system, thigh and shank relative to each other. Synchronisation between the systems was made visually. The reliability in positioning IMU sensors was acceptable for rehabilitation programs due to the shape of the IMU including an alarm when the malalignment was greater than 15° during calibration. Furthermore, results from the validation with use of OTS showed a mean difference of 5° in knee flexion and 3° in thorax flexion in between systems. This discrepancy is higher than the difference between OTS and IMU systems that was observed for knee motion in present study, which did not reach statistical significance.

Bolink et al. compared one single IMU sensor with OTS recordings of pelvic movements during gait in 17 healthy subjects [40]. The error of the IMU system was estimated at 2.7°, which is higher than in this study, although the correlation between the two methods was high (rho = 0.92), which also is in contrast to our observations.

The IMU’s and OTS system use a global coordinate system, but nonetheless the correlation between the data of pelvic movements recorded was poor. It should however be noted that the measured values of pelvic tilt were small. Soft tissue motion around the pelvis generate about the same magnitude of errors as when recording hip and knee flexion. The relative influence of the error when related to the magnitude of the recorded value will therefore be larger than for measurements of hip and knee motions. Another source of error could be that the definition of the pelvic position is based on skin markers initially attached overlying skeletal landmarks. The proximity of the landmark and the IMU sensors may be lost by the time the investigation starts. This will cause inaccuracy in measurements of the rotation of pelvis in the sagittal plane due to different starting positions according to the global coordinate system. Positioning of the IMU sensors on pelvis (left and right) will not take into account the amount of pelvic tilt compared to the OTS in a standing still position. Another difference is that the OTS use one pelvis segment and the IMU system use two separate sensors (left and right) during calculations assuming that left and right pelvis moves independently. The mean value of the two sensors (left and right) was used in the comparison of the two systems. This might allow the range of motion (RoM) to slide relative to one another in sagittal plane. Furthermore, the OTS calculates hip motions relative to the pelvis coordinate system and the knee motions relative to the coordinate system of the thighs. The IMU system calculates the sagittal angles between the segments relative to the global coordinate system defined by an axis of rotation that is not constrained to lie in either the sagittal or frontal planes. It is also important to know that there is a certain amount of movement in the other planes which can generate cross-talk, which at least, to a certain extent could obscure the results. The two systems record motions using alternative algorithms; nevertheless, they show a comparatively high degree of agreement when measuring range of hip and knee motions in the sagittal plane.

## Conclusions

IMUs can produce valid kinematic data of pelvis and knee flexion-extension range. This is in agreement with a previous study using the same IMU system for knee flexion range. The IMUs recorded slightly smaller hip flexion-extension ranges than OTS. Even though there was a significant difference there was a high correlation between the systems on calculations of hip angles. The difference may be due to the difference in the modelling of the pelvis, soft tissue artefacts, misplacements of IMU’s or malalignment of the devices attached to the soft tissues between the two systems. Further studies including recordings in real time for both systems could be interesting to further explore possible reasons for underestimation of values for the range of sagittal plane kinematics with the IMU system.

## Abbreviations

BMI: Body mass index
CI: Confidence interval
ICC: Intraclass correlation coefficient
IMU: Inertial measurement units
OA: Osteoarthritis
OTS: Optical tracking system
PROMs: Patient reported outcome measurement
RoM: Range of motion
RSA: Radiostereometric analysis
SHAR: Swedish Hip Arthroplasty Register
STA: Soft tissue artefacts
THA: Total hip arthroplasty
